# Assessment of Volatile Aromatic Compounds in Smoke Tainted Cabernet Sauvignon Wines Using a Low-Cost E-Nose and Machine Learning Modelling

**DOI:** 10.3390/molecules26165108

**Published:** 2021-08-23

**Authors:** Vasiliki Summerson, Claudia Gonzalez Viejo, Alexis Pang, Damir D. Torrico, Sigfredo Fuentes

**Affiliations:** 1 Digital Agriculture, Food and Wine Group, Faculty of Veterinary and Agricultural Sciences, School of Agriculture and Food, The University of Melbourne, Building 142, Parkville, VIC 3010, Australia; summersonv@unimelb.edu.au (V.S.); cgonzalez2@unimelb.edu.au (C.G.V.); alexis.pang@unimelb.edu.au (A.P.); 2 Department of Wine, Food and Molecular Biosciences, Faculty of Agriculture and Life Sciences, Lincoln University, Lincoln 7647, Canterbury, New Zealand; Damir.Torrico@lincoln.ac.nz

**Keywords:** machine learning, electronic nose, wine quality, climate change, bushfires, artificial neural networks

## Abstract

Wine aroma is an important quality trait in wine, influenced by its volatile compounds. Many factors can affect the composition and levels (concentration) of volatile aromatic compounds, including the water status of grapevines, canopy management, and the effects of climate change, such as increases in ambient temperature and drought. In this study, a low-cost and portable electronic nose (e-nose) was used to assess wines produced from grapevines exposed to different levels of smoke contamination. Readings from the e-nose were then used as inputs to develop two machine learning models based on artificial neural networks. Results showed that regression Model 1 displayed high accuracy in predicting the levels of volatile aromatic compounds in wine (R = 0.99). On the other hand, Model 2 also had high accuracy in predicting smoke aroma intensity from sensory evaluation (R = 0.97). Descriptive sensory analysis showed high levels of smoke taint aromas in the high-density smoke-exposed wine sample (HS), followed by the high-density smoke exposure with in-canopy misting treatment (HSM). Principal component analysis further showed that the HS treatment was associated with smoke aroma intensity, while results from the matrix showed significant negative correlations (*p* < 0.05) were observed between ammonia gas (sensor MQ137) and the volatile aromatic compounds octanoic acid, ethyl ester (r = −0.93), decanoic acid, ethyl ester (r = −0.94), and octanoic acid, 3-methylbutyl ester (r = −0.89). The two models developed in this study may offer winemakers a rapid, cost-effective, and non-destructive tool for assessing levels of volatile aromatic compounds and the aroma qualities of wine for decision making.

## 1. Introduction

Wine aroma is a critical component of wine quality and is determined by the combinations and complex interactions of numerous volatile compounds [1,2,3]. Understanding the aroma character of wine is essential for ensuring quality and consumer acceptance in a competitive market. Hence, identifying the volatile aromatic compounds present in wines can provide valuable information on the sensory attributes of the wine and the viticultural and/or winemaking practices that could be altered to improve the quality [4,5,6] or maintain a certain wine style.

Numerous factors can influence the volatile aromatic compound composition of wines, including environmental conditions, viticultural practices, such as crop-level reduction, and drying of fruit, canopy management, and winemaking practices, such as yeast selection and the use of malolactic bacteria [7,8,9,10]. Furthermore, it has been predicted that the effects of climate change may have profound impacts on the aromatic potential of grapes and hence wine quality [11], particularly the increased risk and incidence of bushfires, resulting in grapevine smoke exposure and smoke taint in wines [12,13,14]. Grapevine exposure to smoke during the critical stages between veraison and harvest has been shown to alter the volatile aromatic composition of grapes and lead to the development of smoke taint in wine, resulting in objectionable smoky characters and reduced wine quality [12,13,14,15,16]. Volatile phenols present in smoke, including guaiacol and 4-methylguaiacol, are responsible for the development of smoke aromas in smoke tainted wines such as burnt wood, burning rubber, medicinal, and smoked meats [13,15,16]. In addition to this, increases in temperature and drought brought on by climate change can also affect the aromatic compounds in grapes and hence wine quality [11,17].

The assessment of the volatile aromatic compound composition of wine typically involves expensive instrumentation such as Gas Chromatography–Mass Spectrometry (GC–MS). However, this form of assessment requires the use of specialised equipment, tedious sample preparation, and trained personnel [18,19,20]. In addition, sensory evaluation using a trained panel is often employed to assess wine quality [5,21]. However, this form of assessment requires the recruitment and training of a large number of participants, which can be expensive and time-consuming, and the results may be subject to bias due to individual variability of the participants, which may affect their taste and smell [5,21,22]. There is, therefore, a need for a rapid, cost-effective method for assessing the volatile aromatic compound and sensory qualities of wine that winemakers can use in the winery [18].

Electronic noses (e-noses) typically consist of an array of gas sensors (e.g., metal oxide semiconductors) coupled with a data processing unit and pattern recognition methods to identify the aroma profiles [12,23,24]. They have been used for numerous applications in the food and beverage industry, including for assessment of geographical origin [23,25], assessing quality, and spoilage [26,27,28], as well as for food safety and adulteration detection [29]. E-noses offer numerous advantages for analysing wine aromatic compounds, including ease of use, rapid results, portability, and non-destructive nature [24,25]. Research by Fuentes et al. [12] found that a low-cost e-nose coupled with machine learning modelling is an effective tool for predicting levels of smoke-derived volatile phenols and their glycoconjugates in smoke-tainted Cabernet Sauvignon wines. Other research by Shim and Lee [30] found a portable and inexpensive e-nose to be effective in classifying French red wines and monitoring wine aging, while research by Han et al. [21] also found a low-cost e-nose and voltammetric tongue effective tools for identifying red wines that differ in geographical origin.

This study explored the use of a low-cost and portable e-nose to assess volatile aromatic compounds and smoke aroma intensity in wines produced from grapes exposed to different densities of smoke exposure with or without in-canopy misting. The e-nose readings were used as inputs for machine learning to develop two artificial neural network (ANN) regression models. Model 1 was constructed using the mean values for the peak areas of the volatile aromatic compounds from GC–MS as targets, while Model 2 was developed using the smoke aroma intensity responses from a trained sensory panel. The two models developed displayed high accuracies in predicting the levels of volatile aromatic compounds and smoke aroma intensity in wine. This can offer winemakers a rapid, cost-effective, and non-destructive tool for assessing the aroma qualities of wine.

## 2. Results and Discussion

### 2.1. GC–MS Analysis

Mean values for the peak areas of the volatile aromatic compounds and their standard errors are shown in Table 1. Significant differences (*p* < 0.05) between different smoke treatments were seen for nonanoic acid, ethyl ester, ethyl 9-decenoate, decanoic acid, ethyl ester, octanoic acid, 3-methylbutyl ester, and dodecanoic acid, ethyl ester. The LS sample showed peaks particularly related to aromatic compounds such as decanoic acid, ethyl ester and dodecanoic acid, ethyl ester. These two compounds are associated with grape, fruity, candy, floral, waxy, oily, and soapy aromas (Table 1). The volatile aromatic compounds observed are fatty acid esters formed during fermentation by yeast, with high levels arising in the LS treatment potentially due to increased fermentation activity. Kennison et al. [15] found that grapevine smoke exposure resulted in increased levels of free amino nitrogen (FAN) in grapes and an increased fermentation rate in grapes exposed to repeated smoke exposure. This increased activity by fermentation yeasts may have resulted in greater levels of fatty acid esters. Furthermore, smoke exposure negatively affects grape ripening, such as sugar accumulation. Previous research also showed that high levels of smoke exposure resulted in increased leaf senescence, which can also impact grape maturation and ripening [15,31]. Therefore, these factors may have reduced the aromatic compound potential of the HS and HSM treatment wines [32,33].

### 2.2. Smoke Aroma Intensity

**Figure 1:**
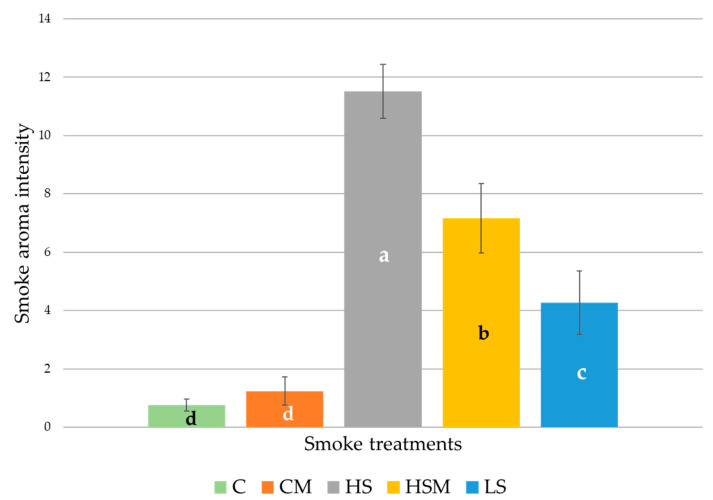
Mean values of smoke aroma intensities as rated using a 15 cm intensity scale with the letters of significance from the ANOVA and Fisher least significant difference (LSD) post hoc test (*p* < 0.05; α = 0.05). Abbreviations: C = control, CM = control with in-canopy misting, HS = high-density smoke exposure, HSM = high-density smoke exposure with in-canopy misting, LS = low-density smoke exposure, SE = standard error.

Figure 1 shows the means and standard error for smoke aroma intensity according to the different smoke treatments. There were significant differences (*p* < 0.05) between samples. As expected, the HS treatment had the highest mean response for smoke aroma intensity (11.52), followed by the HSM treatment (7.17), while the C and CM treatments exhibited the lowest mean value (0.76 and 1.24, respectively). This is in line with previous studies showing the development of smoke aromas following grapevine smoke exposure, with repeated exposure resulting in a cumulative effect on the levels of smoke-derived volatile phenols [13,15,16]. Wines produced from grapes exposed to smoke have been shown to contain higher levels of smoke-derived volatile phenols and exhibit greater smoky characteristics, described as burnt rubber, smoked meat, and disinfectant/hospital [15]. Therefore, there is no surprise that the HS exhibited the highest mean value for smoke aroma intensity, while the two control treatments exhibited the lowest.

### 2.3. Electronic Nose

**Figure 2:**
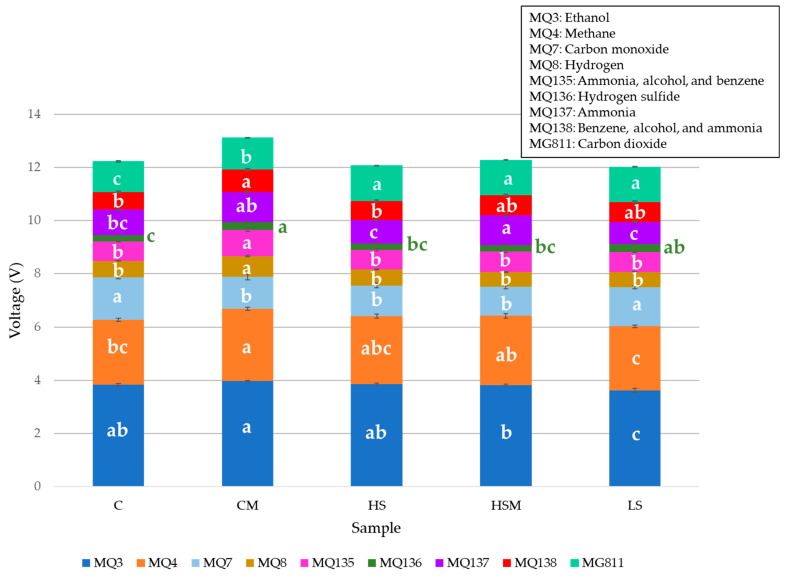
Mean stacked values of the electronic nose depicting the letters of significance from the ANOVA and Fisher least significant difference (LSD) *post hoc* test (*p* < 0.05; α = 0.05). Abbreviations: C = control, CM = control with in-canopy misting, HS-high = density smoke exposure, HSM = high-density smoke exposure with in-canopy misting, LS = low-density smoke exposure.

Figure 2 shows the mean values and standard errors for each gas sensor integrated into the e-nose for each wine sample. Significant differences (*p* < 0.05) were found between wine samples for all gas sensors in the e-nose. The highest readings for all samples were observed for ethanol gas release (sensor MQ3), with the CM treatment exhibiting the highest mean value (3.97 V) and the LS treatment showing the lowest (3.62 V). The lowest readings for all wine samples were seen for hydrogen sulphide gas (sensor 136), with the CM treatment displaying the highest reading again (0.30 V) and the C treatment exhibiting the lowest reading (0.23 V). Hydrogen sulphide (H_2_S) is produced naturally during fermentation by yeast and is responsible for negative aromas in wine, such as rotten eggs, sewage, and cooked cabbage, which can develop after the wine has been bottled [36,37,38,39,40]. Therefore, it is necessary for winemakers to know the concentration of H_2_S in wine and ensure it is kept to a minimum [37]. Readings for the CO_2_ gas sensor (sensor MG811) are inversed; therefore, lower volts mean a higher concentration. The C treatment showed the lowest reading (1.16 V) and, hence, the highest concentration of CO_2_, while the HSM, HS, and LS treatments exhibited the highest readings (1.33 V) and, therefore, the lowest CO_2_ concentration. The CM wines showed the highest mean values for most gases, including ethanol (sensor MQ3), hydrogen (sensor MQ8), and ammonia, alcohol, and benzene (sensor MQ135). As winemaking practices were the same for all the wine samples produced, this may be due to the misting treatment, which may have impacted the grape berries. In particular, misting may have impacted the natural flora present on the grape berries by increasing the humidity surrounding the grape bunch. Therefore, the resulting increase in natural flora may have affected fermentation and the gases produced [41].

### 2.4. Multivariate Data Analysis

**Figure 3:**
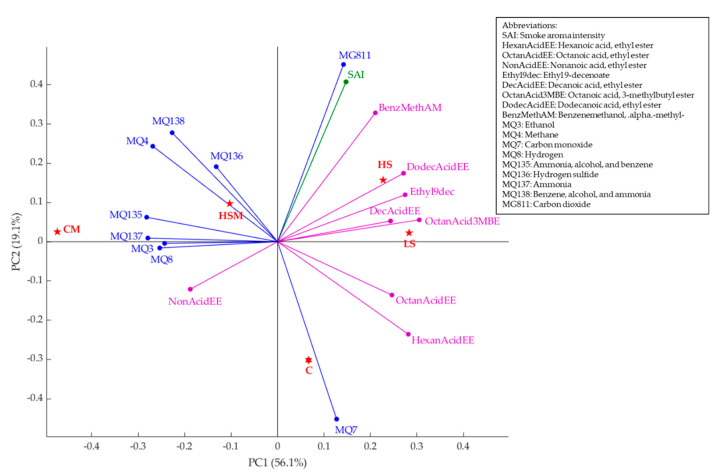
Principal component analysis displaying the e-nose readings (blue), smoke aroma intensity (green) and volatile aromatic compounds (purple). Abbreviations: C = control, CM = control with in-canopy misting, HS = high-density smoke exposure, HSM = high-density smoke exposure with in-canopy misting, LS = low-density smoke exposure.

Figure 3 shows the principal component analysis (PCA) with data from the e-nose readings, volatile aromatic compounds, and smoke aroma intensity. Principal component one (PC1) represented 56.1% of the data variability, and principal component two (PC2) accounted for 19.11%, with a total of 75.20% of the data variability. According to the factor loadings (FL), PC1 was primarily represented by octanoic acid, 3-methylbutyl ester (FL = 0.31), hexanoic acid, ethyl ester (FL = 0.28), ethyl 9-decenoate and dodecanoic acid, ethyl ester (FL = 0.27 for each) on the positive side, and gas sensors MQ135 (FL = −0.28), MQ137 (FL = −0.28), and MQ4 (FL = −0.24) on the negative side of the axis. On the other hand, PC2 was mainly represented by MG811 gas sensor (FL = 0.45), smoke aroma intensity (FL = 0.41) and benzene methanol, alpha-methyl-(FL = 0.33) on the positive side of the axis, and MQ7 gas sensor (FL = −0.45), hexanoic acid, ethyl ester (FL = −0.24), and octanoic acid, ethyl ester (FL = −0.14) on the negative side. It can be observed that the HS and LS wine samples were grouped and associated with volatile aromatic compounds, including dodecanoic acid, ethyl ester, decanoic acid, ethyl ester, octanoic acid, 3-methylbutyl ester, and benzene methanol, alpha-methyl-, in line with the GC–MS results. These results coincide with findings from Summerson et al. [42] in smoke tainted Pinot Grigio wines. Furthermore, HS and LS samples were also associated with smoke aroma intensity and carbon dioxide gas (sensor MG811). The HSM treatment was associated with methane (gas sensor MQ4). According to the MQ4 sensor specifications, it has some sensitivity to smoke, explaining its relationship with the HSM treatment.

On the other hand, the CM treatment was associated with ammonia, alcohol, and benzene (sensor MQ135), and ethanol (sensor MQ3), benzene, alcohol, and ammonia (gas sensor 138), and hydrogen sulphide (sensor MQ136), while the C treatment as associated with carbon monoxide (sensor MQ7). The associations found between CM and the gas sensors mentioned are in accordance with results presented by Summerson et al. [42] for control (non-smoked) Pinot Grigio wines with an amelioration activated carbon treatment.

**Figure 4:**
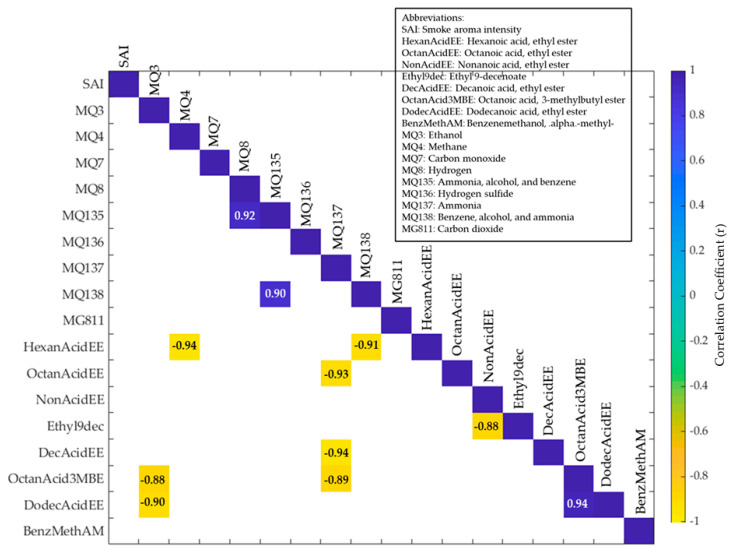
Matrix illustrating the significant (*p* < 0.05) correlations between the sensory parameters, e-nose readings, and volatile aroma compounds. Colour bar: the blue side depicts the positive correlations, while the yellow side depicts the negative correlations. Darker blue and yellow colours denote higher correlations.

Significant correlations (*p* < 0.05) between the sensory parameters, e-nose readings, and volatile aroma compounds are displayed in Figure 4. Positive correlations could be seen between ammonia, alcohol, and benzene gas (sensor MQ135) and hydrogen gas (sensor MQ8) (r = 0.92) and benzene, alcohol, and ammonia gases (sensor MQ138; r = 0.90). This correlation between MQ135 and MQ138 was expected as, even though they have different sensitivity, both can detect ammonia [43]. Negative correlations were observed between ammonia gas (sensor MQ137) and octanoic acid, ethyl ester (r = −0.93), decanoic acid, ethyl ester (r = −0.94) and octanoic acid, 3-methylbutyl ester (r = −0.89), as well as between ethanol gas (sensor MQ3) and octanoic acid, 3-methylbutyl ester (r = −0.88), and dodecanoic acid, ethyl ester (r = −0.90). 

### 2.5. Machine Learning Modelling

**Figure 5:**
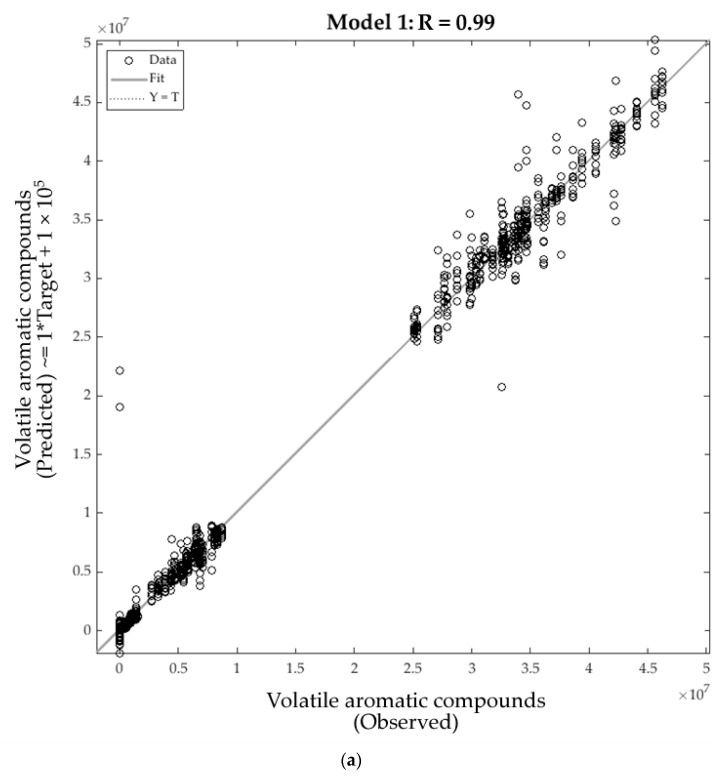

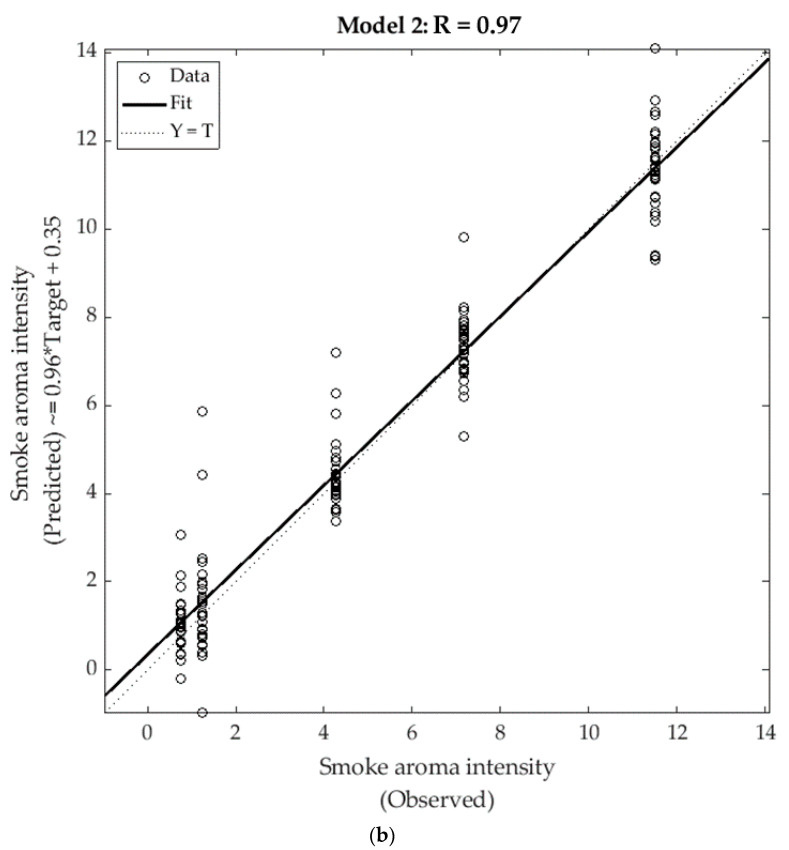
Overall correlations of the two models to predict: (**a**) the levels of volatile aromatic compounds (Model 1) and (**b**) levels of smoke aroma intensities (Model 2).

Table 2 shows the statistical data for the artificial neural network (ANN) regression models developed to predict the levels of volatile aromatic compounds (Model 1) and smoke aroma intensities (Model 2) in the wine samples. Model 1 displayed a high overall correlation and determination coefficients (R = 0.99, R^2^ = 0.98; Figure 5a). Furthermore, there were no signs of over- or underfitting as illustrated by the performance values for the training stage (MSE = 8.39 × 10^12^) being lower than that for the testing stage (MSE = 5.24 × 10^11^). In addition to this, Model 2 also displayed high overall correlation and determination coefficients (R = 0.97, R^2^ = 0.94; Figure 5b), with the performance values for the training stage (MSE = 0.42) once again being lower than that for the testing stage (MSE = 2.76).

The ANN regression models developed in this study displayed high accuracy in predicting the levels of volatile aromatic compounds and smoke aroma intensity in wine. This may offer winemakers cost-effective, rapid tools for assessing the aroma and quality of the wine. Due to the learning capacity of ANN, the models may be further fed with more aromatic volatile compounds to improve and enhance their functionality. Furthermore, as the use of an e-nose is non-destructive, repeated measurements are possible. This may be particularly useful for smoke tainted wines as winemakers can assess the aroma potential of wines before and after applying smoke taint amelioration treatments. Summerson et al. [42] used the e-nose coupled with machine learning modelling to assess the effectivity of smoke taint amelioration treatments in Pinot Grigio wines. Furthermore, Fuentes et al. [12] developed an ANN model using e-nose readings as inputs to predict the levels of 7 volatile phenols and 17 glycoconjugates in wine. The use of these models coupled with the two models developed in this study may provide winemakers with tools to accurately assess the quality of smoke tainted wines in near real-time.

## 3. Materials and Methods

### 3.1. Smoke Treatments and Winemaking

Field trials describing smoke and misting treatments have been previously reported by Szeto et al. [44] and Summerson et al. [31] and consisted of: (i) a control treatment (C; i.e., no smoke exposure or in-canopy misting); (ii) a control treatment with in-canopy misting (CM); (iii) high-density smoke exposure (HS); (iv) high-density smoke exposure with in-canopy misting (HSM); and (v) a low-density smoke exposure (LS). Smoke treatments were applied at approximately seven days post-veraison by pumping smoke derived from the combustion of barley straw into purpose-built tents for one hour. Misting was applied on the same day as the smoking treatments, and it was achieved using a purpose-built sprinkler system that delivered water at 11 L h^−1^ and provided a constant supply of fine water droplets (65 µm) to the grape bunch zone [44,45]. The misting sprinkler system was turned on 5 min before the smoking treatment and turned off 15 min after the treatment; this lasted for a total of 2.5 h because the smoke had to be applied in two sessions (one for each half of vines) as not all vines fitted inside the tent used to isolate smoking. Treatments were applied to six adjacent vines, except for the HSM, which was applied to five adjacent vines, with a minimum of one buffer vine separating all treatments. Once grapes reached maturity, they were harvested for winemaking. The wine was produced on a small scale (~5 kg per fermentation, conducted in triplicate per treatment), as previously described [44].

### 3.2. GC–MS Analysis

Analysis of volatile aromatic compounds was undertaken using a Gas-Chromatograph with a Mass-Selective Detector 5977B (GC-MSD; Agilent Technologies, Inc., Santa Clara, CA, USA) using an HP-5MS column (length 30 m, inner diameter 0.25 mm and film 0.25 µ; Agilent Technologies, Inc., Santa Clara, CA, USA) with helium as the carrier gas (flow rate of 1 mL min^−1^) and an integrated autosampler system PAL3 (CTC Analytics AG, Zwingen, Switzerland). The assessment was conducted in triplicates for each smoke treatment using 5 mL of wine sample that was placed in a 20 mL vial and then analysed using the headspace method with a solid-phase microextraction (SPME) divinylbenzene–carboxen–polydimethylsiloxane (DVB–CAR–PDMS) 1.1 mm grey fibre (Agilent Technologies, Inc.), with a blank vial used at the start to prevent any carryover effects. Additional details about the method used are described by Gonzalez Viejo et al. [46]. Compounds were identified using the National Institute of Standards and Technology (NIST; National Institute of Standards and Technology, Gaithersburg, MD, USA) library, and only compounds with greater than 80% certainty were used for this study.

### 3.3. Assessment of Smoke Aroma Intensity

Sensory evaluation investigating the smoke aroma intensity of the wine samples was conducted in the sensory laboratory of the Faculty of Veterinary and Agricultural Sciences (FVAS). The sensory panel consisted of 13 participants (age: 26–46 years; gender: 69% female and 31% male) from the staff and students at the University of Melbourne (UoM; Ethics ID: 1545786.2). Panellists were previously trained using the quantitative descriptive analysis (QDA^®^) method. The session was undertaken in individual booths equipped with a tablet PC programmed with the Bio-Sensory Application (The University of Melbourne, Parkville, VIC, Australia; [47]), which displayed the questionnaire. All wine samples were assigned a randomly generated 3-digit random code to avoid bias. Participants received 10 mL of each wine sample served at room temperature (~20 °C) in International Standard Wine Tasting Glasses (Bormioli Luigi, Fidenza, Italy). The degree of smoke aroma intensity was rated on a 15-cm intensity scale (absent–intense). Each sample was evaluated independently (monadically), and participants used plain room temperature water and water crackers as palate cleansers.

### 3.4. Electronic Nose

A low cost, portable e-nose developed by the Digital Agriculture, Food and Wine (DAFW) Group from the FVAS of the UoM and comprised of an array of nine gas sensors with sensitivity to different gases was used to assess the wine samples in triplicates, as previously described [12,43]. The nine sensors and the gases they are sensitive to were: (i) MQ3 = ethanol; (ii) MQ4 = methane; (iii) MQ7 = carbon monoxide; (iv) MQ8 = hydrogen; (v) MQ135 = ammonia, alcohol, and benzene; (vi) MQ136 = hydrogen sulphide; (vii) = MQ137 = ammonia; (viii) MQ138 = benzene, alcohol, and ammonia; and (ix) MG811 = carbon dioxide. Measurements were conducted by pouring 100 mL of wine sample into a 500 mL beaker, stirring the liquid once, and placing the e-nose on top, which fully covered the beaker for approximately 1 min to collect the gas readings. The e-nose has some holes in between the sensors to allow airflow and avoid oversaturation of the gases. The e-nose was calibrated for 20–30 s between samples to prevent carryover effects between sample readings.

Data from the e-nose was acquired using a customised code written in MATLAB^®^ R2020a (MathWorks, Inc., Natick, MA, USA) to identify the stable signals from when the e-nose was placed on the beaker containing the sample until just before it was removed. Following this, the data was automatically divided into 10 subdivisions to extract the average values per sensor, as previously detailed by Gonzalez Viejo et al. [22]. The average values were then used as inputs for machine learning modelling.

### 3.5. Statistical Analysis and Machine Learning Modelling

Analysis of variance (ANOVA) was performed using Minitab^®^ version 19.2020.1 (Minitab Inc., State College, PA, USA) for the sensory response towards smoke aroma intensity, e-nose readings, and relative peak areas of the volatile compounds identified by GC–MS, with mean comparisons performed using the Fisher’s least significant difference (LSD) post-hoc test at α = 0.05 to assess if there were significant differences between wine samples. Principal component analysis (PCA) was performed for the sensory response, e-nose readings and volatile aromatic compounds using a customised code written in MATLAB^®^ R2020a, while a matrix was also developed using MATLAB^®^ R2020a to assess the significant correlations (*p* < 0.05) between these parameters.

**Figure 6:**
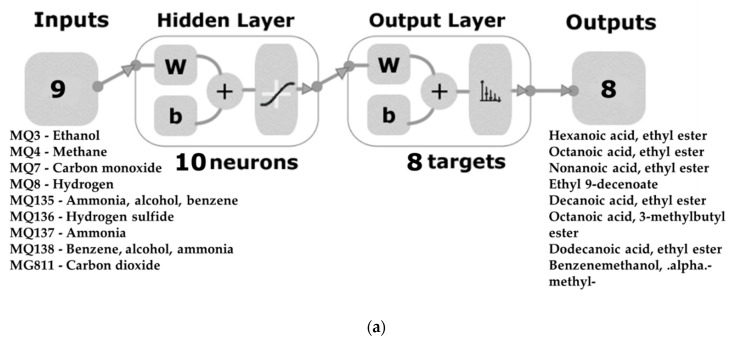

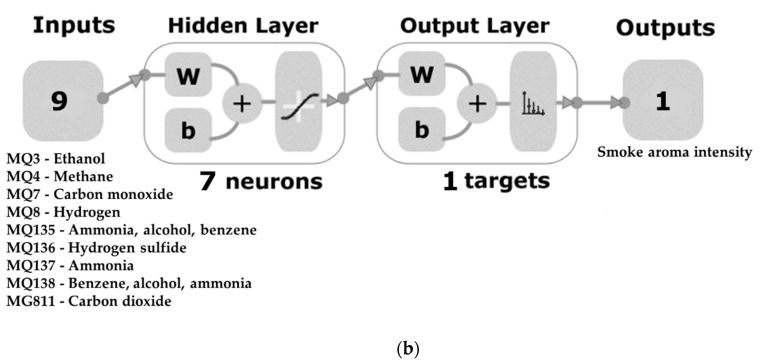
Two-layer feedforward network for the artificial neural network models developed to predict: (**a**) the levels of volatile aromatic compounds present in wine (Model 1) and (**b**) smoke aroma intensity (Model 2). Abbreviations: W = weights, b = biases.

Machine learning modelling based on ANN was performed to develop the regression models using a customised code written in MATLAB^®^ R2020a that tested 17 different training algorithms, as Gonzalez Viejo et al. [48] described previously. The 10 mean values of each of the e-nose outputs for each replicate of the five smoke treatments were used as inputs to predict levels of: (i) the eight volatile aromatic compounds present in the wine samples (Model 1; Figure 6a), and (ii) the level of smoke aroma intensity (Model 2; Figure 6b). The Bayesian Regularisation algorithm was the best for both models based on the high accuracy and performance observed, with further training performed to develop more accurate ANN models, with no signs of under- or overfitting. Input data were divided randomly, with 80% used for training and 20% used for testing for each model, with performance tested based on mean squared error (MSE). Ten neurons were used for Model 1, while seven were used for Model 2 following a trimming test with 3, 7, and 10 neurons (data not shown).

## 4. Conclusions

The use of a low-cost, portable electronic nose coupled with machine learning may offer winemakers a more cost-effective and rapid tool for assessing levels of volatile aromatic compounds and the degree of smoke aroma intensity in wine. Furthermore, the non-destructive nature of this form of assessment allows for repeated measurements, allowing winemakers to assess the quality of wine samples before and after the application of smoke-taint amelioration treatments in smoke tainted wines. The ANN regression models may also be used alongside previously developed models that assess the levels of smoke derived volatile phenols and their glycoconjugates and consumer sensory responses towards wine samples for an in-depth quality assessment of smoke-tainted wines.

## Figures and Tables

**Table 1:** Aromatic compounds detected from the GC–MS analysis showing their odour description, mean peak area (top), and standard error (bottom).

Compound	RT	RI	Odour Description	C	CM	HS	HSM	LS
Hexanoic acid, ethyl ester (ns)	12.15	996	Fruity, apple, sweetish, spicy [1,2,3,34]	6.72 × 10^6^	4.97 × 10^6^	6.29 × 10^6^	5.65 × 10^6^	6.47 × 10^6^
				±8.19 × 10^5^	±1.66 × 10^6^	±3.07 × 10^5^	±3.60 × 10^5^	±1.09 × 10^6^
Octanoic acid, ethyl ester (ns)	16.25	1196	Apple, fruity, sweetish, floral [2,3,34]	4.07 × 10^7^	3.57 × 10^7^	4.16 × 10^7^	3.41 × 10^7^	4.08 × 10^7^
				±1.69 × 10^6^	±5.46 × 10^6^	±5.54 × 10^5^	±1.99 × 10^6^	±3.49 × 10^6^
Nonanoic acid, ethyl ester	18.02	1294	Fruity, nutty, floral [1,34]	3.57 × 10^5 a^	6.27 × 10^5 a^	0 ^b^	4.37 × 10^5 a^	5.13 × 10^5 a^
				±1.79 × 10^5^	±9.82 × 10^4^	±0	±3.72 × 10^4^	±3.27 × 10^4^
Ethyl 9-decenoate	19.57	1387.8	Fruity, fatty [35]	1.04 × 10^6 b^	6.98 × 10^5 c^	1.44 × 10^6 a^	9.07 × 10^5 bc^	1.13 × 10^6 ab^
				±8.22 × 10^4^	±1.54 × 10^5^	±4.96 × 10^4^	±4.47 × 10^4^	±1.13 × 10^5^
Decanoic acid, ethyl ester	19.70	1373	Grape, oily [1,2,3,34]	3.01 × 10^7 b^	2.88 × 10^7 b^	3.21 × 10^7 ab^	2.79 × 10^7 b^	3.51 × 10^7 a^
				±1.24 × 10^6^	±2.62 × 10^6^	±1.11 × 10^6^	±1.43 × 10^6^	±7.82 × 10^5^
Octanoic acid, 3-methylbutyl ester	20.51	1450.4	Sweet, oily, fruity, soapy, pineapple, coconut [35]	4.64 × 10^5 ab^	2.95 × 10^5 b^	5.44 × 10^5 a^	4.35 × 10^5 ab^	6.31 × 10^5 a^
				±2.47 × 10^4^	±1.49 × 10^5^	±3.48 × 10^4^	±1.94 × 10^4^	±2.98 × 10^4^
Dodecanoic acid, ethyl ester	22.75	1597	Candy, floral, fruity, waxy, soap [1,34]	4.58 × 10^6 c^	2.67 × 10^6 d^	6.49 × 10^6 b^	6.16 × 10^6 b^	8.31 × 10^6 a^
				±1.19 × 10^5^	±7.20 × 10^5^	±3.78 × 10^5^	±3.54 × 10^5^	±2.42 × 10^5^
Benzene methanol, alpha-methyl-(ns)	14.62	1194	Chemical, medicinal, naphthyl, gardenia, hyacinth [35]	2.13 × 10^7^	2.39 × 10^7^	3.33 × 10^7^	2.44 × 10^7^	3.40 × 10^7^
				±1.07 × 10^7^	±1.20 × 10^7^	±7.12 × 10^5^	±1.22 × 10^7^	±6.25 × 10^5^

Abbreviations: RT = retention time; C = control; CM = control with in-canopy misting; HS = high-density smoke exposure; HSM = high-density smoke exposure with in-canopy misting; LS = low-density smoke exposure; ns = not significant. Means followed by different letters within each column are statistically significant based on Fisher’s least significant difference (LSD) post hoc test (α < 0.05).

**Table 2:** Statistical results for the artificial neural network models developed to estimate the levels of volatile aroma compounds (Model 1) and smoke aroma intensity (Model 2) in wine, showing the correlation coefficient (R), determination coefficient (R^2^), slope (b), and performance based on mean squared error (MSE) for each stage.

Stage	Samples	Observations	R	R^2^	b	Performance (MSE)
Model 1						
Training	240	1920	0.99	0.98	1.00	8.39 × 10^12^
Testing	60	480	0.98	0.96	1.00	5.24 × 10^11^
Overall	300	2400	0.99	0.98	1.00	-
Model 2						
Training	240	240	0.99	0.98	0.96	0.42
Testing	60	60	0.94	0.88	0.95	2.76
Overall	300	300	0.97	0.94	0.96	-

- Not applicable.

## Data Availability

Data and intellectual property belong to The University of Melbourne; any sharing needs to be evaluated and approved by the university.

## Sample Availability

Samples of the compounds are not available.
